# Clinicopathological characteristics of progressive gastrointestinal stromal tumors and heterogeneity analyses of secondary mutations

**DOI:** 10.1093/oncolo/oyaf110

**Published:** 2025-05-16

**Authors:** Jiaxin Li, Lin Sun, Shasha Liu, Huimin Liu, Bin Li, Hongjie Zhan, Yan Sun

**Affiliations:** Department of Pathology, Tianjin Medical University Cancer Institute & Hospital, National Clinical Research Center for Cancer, Tianjin’s Clinical Research Center for Cancer, Tianjin Key Laboratory of Digestive Cancer, Tianjin 300060, People’s Republic of China; Department of Pathology, Tianjin Medical University Cancer Institute & Hospital, National Clinical Research Center for Cancer, Tianjin’s Clinical Research Center for Cancer, Tianjin Key Laboratory of Digestive Cancer, Tianjin 300060, People’s Republic of China; Department of Pathology, Tianjin Medical University Cancer Institute & Hospital, National Clinical Research Center for Cancer, Tianjin’s Clinical Research Center for Cancer, Tianjin Key Laboratory of Digestive Cancer, Tianjin 300060, People’s Republic of China; Department of Pathology, Tianjin Medical University Cancer Institute & Hospital, National Clinical Research Center for Cancer, Tianjin’s Clinical Research Center for Cancer, Tianjin Key Laboratory of Digestive Cancer, Tianjin 300060, People’s Republic of China; Department of Gastric Surgery, Tianjin Medical University Cancer Institute & Hospital, National Clinical Research Center for Cancer, Tianjin’s Clinical Research Center for Cancer, Tianjin Key Laboratory of Digestive Cancer, Tianjin 300060, People’s Republic of China; Department of Gastric Surgery, Tianjin Medical University Cancer Institute & Hospital, National Clinical Research Center for Cancer, Tianjin’s Clinical Research Center for Cancer, Tianjin Key Laboratory of Digestive Cancer, Tianjin 300060, People’s Republic of China; Department of Pathology, Tianjin Medical University Cancer Institute & Hospital, National Clinical Research Center for Cancer, Tianjin’s Clinical Research Center for Cancer, Tianjin Key Laboratory of Digestive Cancer, Tianjin 300060, People’s Republic of China

**Keywords:** gastrointestinal stromal tumor (GIST), progressive, secondary mutation, heterogeneity

## Abstract

**Background:**

Although there have been multiple lines of drugs for gastrointestinal stromal tumors (GISTs), the drug response depends on the progressive tumors’ biological behaviors and secondary mutations.

**Methods:**

We investigated the primary and secondary mutations in multiple tumors from the same patients and at multiple regions from the same tumor to analyze the inter- and intratumoral heterogeneities using Sanger sequencing and next-generation sequencing (NGS).

**Results:**

Secondary mutations were more frequently detected in patients with a targeted therapy history and who continued their targeted therapy until surgery or biopsy, in larger tumors, and in tumors located in the intestine, abdominal cavity, and mesentery. Secondary mutations were detected in only 57.5% of the samples from the cases with secondary mutations, and 34.8% of the cases presented multiple types of secondary mutations, including both intertumoral and intratumoral heterogeneities. Temporal heterogeneity was also observed at different time points of progression. The results of NGS and Sanger sequencing were consistent for the individual sample, but Sanger sequencing detected multiple types of secondary mutations from different tumors of the same patient. Liquid biopsy also only detected partial secondary mutations revealed by Sanger sequencing.

**Conclusion:**

Progressive GISTs had intertumoral and intratumoral heterogeneities of secondary mutations. Sanger sequencing had its own advantage in revealing the heterogeneity of secondary mutations. The improvement in the detection rate of secondary mutations by selecting the appropriate tumor sample to be tested, or even the appropriate tumor region or test method, is helpful to identify the optimal drugs for progressive GISTs.

**Implications for practice**

Our study underscored the clinicopathologic characteristics of progressive GISTs at the time of initial diagnosis and progression, contributing to finding the patients with a high risk of disease progression and accordingly performing a close follow-up and timely treatment. In addition, we demonstrated the intertumoral, intratumoral, and temporal heterogeneities of secondary mutations in progressive GISTs. The improvement in the detection of secondary mutations is helpful to suggest optimal drugs for progressive GISTs through selecting an appropriate tumor sample, even the appropriate tumor region, and a suitable test method.

## Introduction

*KIT*/*PDGFRA*-selective targeted inhibitors have been developed and applied to the treatment of gastrointestinal stromal tumors (GISTs).^1,2^ Imatinib emerged as the first-line treatment for GISTs, except for those with *PDGFRA* D842V mutation, for which Avapritinib was the first-line treatment. Furthermore, other tyrosine kinase inhibitor (TKI) drugs, such as sunitinib, regorafenib, or ripretinib, can be used in patients with progressive tumors after imatinib treatment.^3^ However, the heterogeneity of TKI resistance continues to bring about a substantial clinical challenge.^3^ The drug response depends on the biological behaviors and secondary mutations of the progressive tumors.^4,5^ Therefore, we need to better understand the clinicopathological characteristics and mutation status of these progressive tumors to determine the appropriate drugs and prolong the patients’ survival time. Clinically, some progressive GISTs present multiple tumors, in which secondary mutations may not be detected due to tumor heterogeneity. It remains unclear which patient, or which tumor secondary mutations could be detected more easily.

In the present study, we explored the important parameters related to tumor progression by analyzing the clinicopathological features and mutation status of the patients with progressive tumors at the time of the initial diagnosis and during tumor progression. Moreover, we aimed to analyze the primary and secondary mutations in multiple progressive tumors from the same patients and at multiple regions from the same tumor progressive tumor to understand the intertumoral and intratumoral heterogeneities of these tumors and improve the positivity rate of detecting secondary mutations.

## Methods

### Patient selection

We selected 69 patients with progressive GISTs who underwent operations or biopsy procedures for progressive tumors at Tianjin Medical University Cancer Institute and Hospital from June 2003 to March 2023. We collected 80 tumor tissue samples from the 69 patients (61, 5, and 3 patients had tumor progression once, twice, and third times, respectively), including 10 biopsy and 70 resected tissue samples. The clinicopathological and primary molecular variation data at the time of the initial diagnosis were collected. The present study was approved by the Institutional Review Board of Tianjin Medical University Cancer Institute and Hospital.

### Tumor selection

Altogether, 829 hematoxylin and eosin (H&E)-stained sections were reviewed for sample selection. For single progressive tumors, we selected one sample every 5 cm, and no more than five samples were selected for one tumor. For multiple progressive tumors, all the tumors with diameters of >1 cm were selected. If the multiple progressive tumors were located at one anatomical site, at least 2 tumors were selected. If the tumors were located at different anatomical sites, at least 1 sample from each site was selected.

### Extraction and quantification of DNA

For each sample, DNA extraction was performed based on the H&E sections, excluding sections with hemorrhage, necrosis, or collagenous degeneration. For the tumors with obvious differences in cell density in the H&E sections, we took different regions individually for DNA extraction. DNA was extracted with a QIAamp DNA FFPE Tissue Kit according to the manufacture’s protocol (Qiagen). Blood was collected in Cell-Free DNA BCT tubes (Streck), and plasma and normal white blood cells were processed as previously described.^6^ Circulating free DNA (cfDNA) was isolated from 2 to 4 mL (average 2.2 mL) plasma using a QIAamp Circulating Nucleic Acid Kit (Qiagen). Normal genomic DNA from white blood cells was isolated using a QIAamp DNA Blood Mini Kit (Qiagen). Genomic DNA was quantified using Nanodrop 2000 (Thermo Fisher Scientific), and genomic DNA and cfDNA were quantified using a Qubit fluorometer from Invitrogen (Thermo Fisher Scientific).

### Sanger and next-generation sequencing (NGS)

Sanger sequencing was performed for all samples to evaluate *KIT* exons 9, 11, 13, 14, 17, and 18 and *PDGFRA* exons 12, 14, and 18. The experimental procedures had been described in detail previously.^7^ The Sanger sequencing primers are shown in Supplementary Table S1. Additionally, NGS was performed in seven samples according to the experimental procedures described elsewhere.^7^

### Statistical analyses

Statistical analysis was performed with SPSS 22.0. The overall differences were determined by the chi-square and Bonferroni’s tests for the categorical data of the independent samples. Independent sample *t-*tests, Mann-Whitney *U* tests, and one-way analysis of variance with the least significant difference post hoc tests were used for the continuous variables of the independent samples. A *P* value < .05 was considered to indicate a significant difference.

## Results

### Clinicopathological characteristics of the patients with progressive GIST at the time of the initial diagnosis and during disease progression

When we analyzed the clinicopathologic characteristics of the progressive tumors, we selected the first progressive tumors that developed among patients with multiple progressive tumors to match those samples obtained from patients with only one tumor progression. Among the 69 patients with progressive GIST, the most common progressive site was in situ (*n* = 23), followed by the liver (*n* = 18), mesentery (*n* = 14), abdominal and pelvic cavities (*n* = 13), peritoneum (*n* = 11), retroperitoneum (*n* = 10), abdominal wall (*n* = 9), adjacent structures (*n* = 6), colorectum (*n* = 4), chest cavity/pleura (*n* = 2), and small intestine (*n* = 1). Progressive GISTs involving the mesentery occurred more frequently in patients with primary tumors in the small intestine than in those with primary tumors in the stomach and other sites (32.3% vs 14.8% vs 0.0%, *P *= .019). Progressive GISTs involving the retroperitoneum occurred more frequently in the patients with a targeted therapy history than in those without such a treatment history (19.6% vs 0.0%, *P *= .010). Progressive GISTs involving the retroperitoneum and pelvic cavity occurred more frequently in the patients continuing targeted therapy until surgery or biopsy than in those discontinuing targeted therapy (20.0% vs 3.4%, *P *= .034 and 28.6% vs 6.9%, *P *= .021). Additionally, although not significantly different, all the cases involving the mesentery carried the *KIT* exon 11 deletion mutation but not the missense or insertion mutations (22.9% vs 0.0% vs 0.0%, *P *= .087).

We further analyzed the clinicopathologic characteristics of the 69 patients at the time of the initial diagnosis and compared them between the patients with a single progressive tumor and those with multiple progressive tumors. As shown in Table 1, the patients with primary tumors in the small intestine, with an intermediate risk, and with *KIT* exon 9 insertion mutations more frequently presented with multiple progressive tumors than those with primary tumors in the stomach (*P *= .023), with other risk degrees (*P *= .034), and with *KIT* exon 11 mutations (*P *= .017), respectively. Additionally, multiple progressive tumors are more frequently observed in the liver, abdominal wall, mesentery, and peritoneum (*P *= .013, *P* = .005, *P* = .010, and *P* = .002, respectively).

**Table 1.** The clinicopathological characteristics of 69 patients with progressive GISTs at the time of the initial diagnosis and during disease progression.

Characteristic	All patients (*n* = 69)	Multiple tumors (*n* = 46)	Single tumor (*n* = 23)	*P* value
Gender Male Female	41 (59.4) 28 (40.6)	27 (58.7) 19 (41.3)	14 (60.9) 9 (39.1)	.862
Age at initial diagnosis Median (Min-Max)	56 (17–77)	56 (17–69)	53 (27–77)	.570
Primary site Stomach^§^ Small intestine^§^ Others	27 (39.1) 31 (44.9) 11 (15.9)	14 (30.4) 26 (56.5) 6 (13.0)	13 (56.5) 5 (21.7) 5 (21.7)	.023
Risk for primary tumor Very low Low^§^ Intermediate^§^ High	1 (1.6) 6 (9.7) 18 (29.0) 37 (59.7)	1 (2.4) 2 (4.8) 16 (38.1) 23 (54.8)	0 (0.0) 4 (20.0) 2 (10.0) 14 (70.0)	.034
Primary mutation *KIT* exon9^§^ *KIT* exon11^§^ *KIT* exon13 *PDGFRA* D842V Wild Type	11 (15.9) 46 (66.7) 1 (1.4) 3 (4.3) 8 (11.6)	11 (23.9) 26 (56.5) 1 (2.2) 2 (4.3) 6 (13.0)	0 (0.0) 20 (87.0) 0 (0.0) 1 (4.3) 2 (8.7)	.017
Primary *KIT* exon11 variant Deletion Missense Insertion	35 (76.1) 10 (21.7) 1 (2.2)	20 (76.9) 6 (23.1) 0 (0.0)	15 (81.3) 4 (18.8) 1 (5.0)	.423
Targeted therapy history No Yes	18 (26.1) 51 (73.9)	15 (32.6) 31 (67.4)	3 (13.0) 20 (87.0)	.069
Targeted therapy continued until surgery or biopsy^*^ No Yes	29 (45.3) 35 (54.7)	20 (45.5) 24 (54.5)	9 (45.0) 11 (55.0)	.973
Progression site In site Liver Abdominal cavity Retroperitoneal Abdominal wall Small intestinal Colorectal Pelvic cavity Mesentery Peritoneum Adjacent structures Chest cavity/pleura	23 (33.3) 18 (26.1) 13 (18.8) 10 (14.5) 9 (13.0) 1 (1.4) 4 (5.8) 13 (18.8) 14 (20.3) 11 (15.9) 6 (8.7) 2 (2.9)	16 (34.8) 16 (34.8) 10 (21.7) 5 (10.9) 9 (19.6) 1 (2.2) 3 (6.5) 11 (23.9) 13 (28.3) 11 (23.9) 5 (10.9) 1 (2.2)	7 (30.4) 2 (8.7) 3 (13.0) 5 (21.7) 0 (0.0) 0 (0.0) 1 (4.3) 2 (8.7) 1 (4.3) 0 (0.0) 1 (4.3) 1 (4.3)	.718 .013 .372 .227 .005 .366 .709 .108 .010 .002 .339 .622

^§^: The Bonferroni correction for a chi-square analysis showed a significant difference in this parameter between the patients with multiple progressive tumors and those with a single progressive tumor. ^*^: Five cases were not included due to missing information on the termination of targeted therapy.

### Assessment of secondary mutations in progressive GISTs

The mutations in *KIT* and *PDGFRA* were tested in 335 samples from 69 patients. Among them, the mutation results were unavailable in 129 samples, including 71 samples from 13 patients with poor DNA quality and 58 samples from 10 patients with insufficient DNA quantity. Finally, 206 samples from 56 patients had available mutation results, with each patient having an average of three samples for evaluation (range: 1–17). The detailed results of these 206 samples, along with their clinicopathological information and primary mutation status, are listed in Supplementary Table S2.

The secondary mutations need to be detected in the progressive tumors but not in the primary tumors. Concurrently, the presence of primary mutations, similar to the primary tumor, needs to be determined in the progressive tumors to exclude testing mistakes. In addition, the data on secondary mutations were compared with the data in the COSMIC database (https://cancer.sanger.ac.uk/cosmic) or PubMed to identify any new mutations. According to the abovementioned standards, secondary mutations were detected in 41.1% (23/56) of patients. Among the 23 patients with secondary mutations, a total of 113 samples were tested and secondary mutations were found in 65 (57.5%) samples. We compared the secondary *KIT* mutations in our cohort with those described in previous literature^8-18^ (Table 2). Both *KIT* exon 13 V654A and exon 14 T670I were detected in almost all studies on GIST secondary mutations, whereas different types of secondary mutations in *KIT* exons 17 and 18 were reported in different studies. Although C809, D816, D820, N822, and Y823 were the most common secondary mutation sites, different amino acid changes were detected at these sites in different studies. C809G, D820H/Y/V/G, S821Y, N822K, Y823D, and A829P were detected in both our cohort and those of some previous studies, whereas D816E/G/H, D820E/N, and N822D were not detected in our cohort, but they were reported in some previous studies. Contrarily, we detected some rare types of substitution missense mutations, such as N822I, Y823S, and K826N. Interestingly, albeit it was reported that almost all of the secondary mutations were substitution missense mutations,^12,13,17,19^ we detected the *KIT* exon 17 insertion mutation S821_N822insR, which has not been recorded in the COSMIC database.

**Table 2.** Summary of secondary KIT mutations in progressive GISTs in our cohort and reported in previous literature.

References	No. of cases	Proportion of multiple tumors	Sample Type	No. of test samples	Secondary mutation rate in tested cases	Rate of multiple mutations	Secondary mutation types
Miselli et al^8^	8	37.5% (3/8)	FFPE	20	62.5% (5/8)	NR	V654A, T670I, D820N
Nishida et al^9^	8	12.5% (1/8)	fresh tissue	9	87.5% (7/8)	14.3% (1/7)	V654A, T670I, D816H, N822K, Y823D
Desaiet al^10^	10	NR	FFPE	10	80.0% (8/10)	0.0%	V654A, C809G, D816H, N822K, Y823D,
Lim KH et al^11^	12	66.7% (8/12)	FFPE	15	75.0% (9/12)	22.2% (2/9)	V643ins, V654A, D820G/Y, N822K
Liegl et al^12^	14	85.7% (12/14)	FFPE	57	57.1% (8/14)	75.0% (6/8)	V654A, T670I, D816H, D820G, N822K/Y, Y823D, A829P
Antonescu et al^13^	18	38.9% (7/18)	fresh tissue	18	38.9% (7/18)	0.0%	V654A, T670I, D820Y, N822K, Y823D
Nishida, et al1^4^	25	76.0% (19/25)	fresh tissue	45	80.0% (20/25)	30.0% (6/20)	V654A, T670I, K786N, C809G, D816H/E, D820V, N822K/Y/D, Y823D, A829P
Debiec–Rychter, et al^15^	26	NR	fresh tissue	26	46.2% (12/26)	0.0%	V654A, T670I, D716N, D816G, D820E/Y, N822K
Wardelmann et al^16^	32	NR	FFPE	104	43.8% (14/32)	28.6% (4/14)	V654A, S709F, T670I/E, D816E, D820G/E/Y, N822K, Y823D
Heinrich et al^17^	33	0%	fresh tissue or FFPE	33	66.7% (22/33)	4.5% (1/22)	V654A, T670I, C809G, D816H, D820A/G, N822K, Y823D
Du et al^18^	320	NR	FFPE	320	70.0% (224/320)	0%	V654A, T670I, C809G, D816H, D820Y, N822K/Y, Y823D, A829P
This study	56	64.3% (36/56)	FFPE	206	41.1% (23/56)	34.8% (8/23)	V654A, T670I, C809G, D820G/H/Y/V, S821Y, S821_N822insR, N822I/K, Y823D/S, K826N, A829P

Abbreviations: FFPE, formalin-fixed paraffin-embedding; NR, no record.

### Comparison of the clinicopathological and molecular characteristics between progressive GISTs with and without secondary mutations

We compared the clinicopathological characteristics of the progressive GISTs with and without secondary mutations (only accounting for the first progressive tumors, Table 3). Primary *KIT* exon 11 mutation was more frequently observed in the progressive GISTs with secondary mutations than in those without secondary mutations (*P *= .004). The patients with GISTs with secondary mutations were more likely to have a history of targeted therapy or to continue their targeted therapy until surgery or biopsy than those without secondary mutations (*P *< .001 and *P *< .001, respectively). There were no differences in the male-to-female ratio, age, number of primary tumors, primary sites, risk degree of the primary tumor, and rates of primary *KIT* exon 9 mutation, *PDGFRA* D842V mutation, or *KIT* exon 11 variant type between patients with GISTs with and without secondary mutations.

**Table 3.** Comparison of the clinicopathological and molecular characteristics between progressive GISTs with and without secondary mutations.

Characteristic	With secondary mutations (*n* = 23)	Without secondary mutation (*n* = 33)	*P* value
Gender Male Female	17 (73.9) 6 (26.1)	17 (51.5) 16 (48.5)	.091
Age at initial diagnosis Median (min-max)	55 (38-69)	56 (17-77)	.593
Primary site Stomach Small intestine Others	10 (43.5) 10 (43.5) 3 (13.0)	15 (45.5) 12 (36.4) 6 (18.2)	.813
Risk for primary tumor Very low Low Median High	0 (0.0) 2 (11.1) 4 (22.2) 12 (66.7)	1 (3.2) 2 (6.5) 8 (25.8) 20 (64.5)	.735
Primary mutation * KIT* Exon9 *KIT* Exon11^§^ *KIT* Exon13 *PDGFRA* D842V Wild Type^§^	2 (8.7) 20 (87.0) 1 (4.3) 0 (0.0) 0 (0.0)	7 (21.2) 17 (51.5) 0 (0.0) 3 (9.1) 6 (18.2)	.004
Primary *KIT* Exon11 variant Deletion Missense Insertion	18 (90.0) 1 (5.0) 1 (5.0)	12 (70.6) 5 (29.4) 0 (0.0)	.072
Targeted therapy history No Yes	0 (0.0) 22 (100.0)	12 (36.4) 21 (63.6)	<.001
Targeted therapy continued until one week before surgery^*^ No Yes	1 (4.8) 20 (95.2)	21 (67.7) 10 (32.3)	<.001
Number of tumors at progression Single Multiple	9 (39.1) 14 (60.9)	11 (33.3) 22 (66.7)	.656

^§^: The Bonferroni correction for a chi-square analysis showed a significant difference in this parameter between the patients with and without secondary mutations. ^*^: Three cases were not included due to missing information on the termination of targeted therapy.

We further analyzed the progressive tumors with secondary mutations. Among the 23 patients with secondary mutations, 15 showed one type of secondary mutation and 8 presented with multiple types of secondary mutations, with 6 patients showing 2 mutation types, 1 with 3 mutation types, and 1 with 4 mutation types. The positivity rate of secondary mutations was higher in the patients with multiple types of secondary mutations than in those with one type of secondary mutation (68.3% vs 45.3%, *P *= .013). Moreover, the positivity rate of secondary mutations was different in the diverse progression sites (*P *< .001). Progressive tumors in the lung, chest wall, chest cavity, and pleura had the highest ratio of secondary mutations (80.0%), followed by those in the small intestine (71.4%), abdominal cavity (63.2%), and mesentery (51.4%); progressive tumors in the peritoneum had the lowest ratio of secondary mutations (8.3%). Additionally, the positivity rate of secondary mutations was higher in patients with large progressive tumors than in those with small progressive tumors (5.2 vs. 3.9 cm, *P *= .001).

### Heterogeneity analysis of secondary mutations

**Figure 1.**
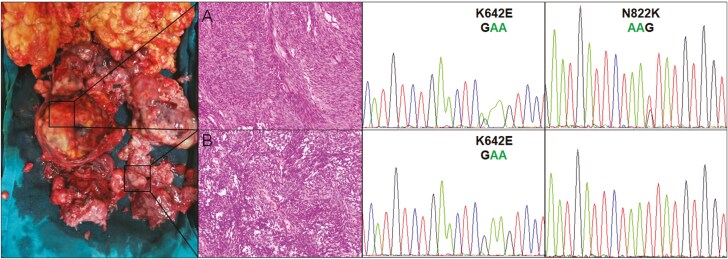
A typical case showing intertumoral heterogeneity in *KIT* mutations (Case 8 in Supplementary Table S2). *KIT* exons 9, 11, 13, 14, 17, and 18 were tested in 14 progressive tumor samples from the peritoneum. A and B were from different tumors. The primary *KIT* exon 13 K642E (AAA > GAA) mutation was detected in all tumors. The secondary N822K (AAT > AAG) mutation was detected in A but not in B. Although none of the secondary mutations were detected, the primary *KIT* exon 13 K642E (AAA > GAA) mutation was almost homozygous in B.

Heterogeneity of secondary mutations occurred among the different tumors (intertumoral heterogeneity) and in the same tumor (intratumoral heterogeneity). Among the 15 patients with one type of secondary mutation, the secondary mutation was observed in all samples in nine patients and in the partial tumors in the other 6 patients (Supplementary Table S2). Interestingly, in Case 8, the secondary *KIT* exon 17 N822K mutation was detected only in one sample obtained from the peritoneum (Figure 1A), but the evolution from heterozygous to homozygous of the primary *KIT* exon 13 K642E mutation was found in the other samples obtained from the abdominal wall, peritoneum, and retroperitoneum (Figure 1B). Although most *KIT*-mutated GISTs were heterozygous,^2^ the evolution from the heterozygous to homozygous *KIT* mutation has been reported to be related to malignant behaviors and a lower sensitivity to imatinib.^20,21^ Among the eight patients with multiple types of secondary mutations, secondary mutations were detected in all samples in five patients, and different secondary mutations were detected in the partial samples in three patients (Supplementary Table S2).

**Figure 2.**
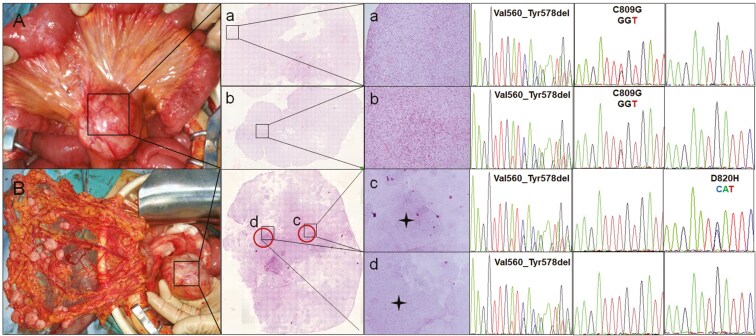
A typical case showing intratumoral heterogeneity in *KIT* mutations (Case 12 in Supplementary Table S2). *KIT* exons 9, 11, 13, 14, 17, and 18 were tested in 10 tumor samples from the mesentery (A) and pelvic cavity and omentum majus (B). A: There was no obvious heterogeneity in either the cell morphology or cell density in the mesenteric mass. Two samples from the different regions (a and b) in one tumor were tested individually. Both the primary V560_Y578del and secondary C809G (TGT > GGT) mutations were detected in both regions. B: The cell density was heterogeneous in the pelvic mass. The regions with dense cells (red circle) from regions c and d were tested individually. The other secondary mutation, D820H (GAT > CAT), but not C809, was detected in region c, whereas no secondary mutation was detected in region d.

Intratumoral heterogeneity was also observed in our study. In a typical patient (Case 12), all 16 samples from progressive tumors presented primary *KIT* exon 11 V560_Y578 deletion mutation, but only 5 samples from the mesenteric mass contained *KIT* exon 17 C809G secondary mutation (Figure 2A), indicating an intertumoral heterogeneity. Moreover, we observed the heterogeneity of cell density in the same pelvic mass. Four dense cell regions from the same tumor were tested individually, of which only one sample had a *KIT* exon 17 D820H secondary mutation (Figure 2B), indicating an intratumoral heterogeneity.

**Figure 3.**
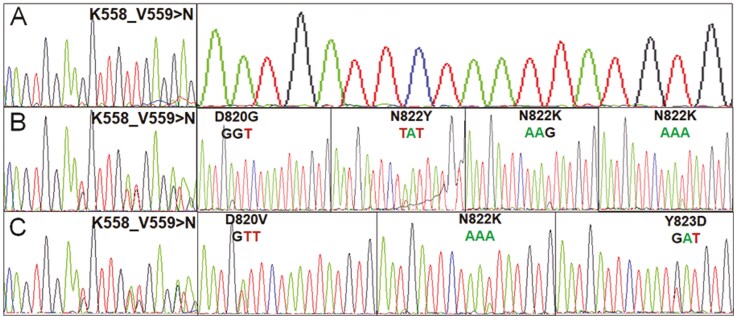
A typical case showing temporal heterogeneity in *KIT* mutations among three progressive tumors at different times of progression (Case 49 in Supplementary Table S2). *KIT* exons 9, 11, 13, 14, 17, and 18 were tested. A. No secondary mutation was detected in the first progressive retroperitoneal tumor (sample 49a) at 61 months after diagnosis; however, primary K558_V559 > N mutation was observed. B. Four types of secondary mutations were detected in six progressive tumor samples from the abdominal cavity (sample 49b), including D820G (in one sample), N822Y (in two samples), N822K (AAT > AAG, in one sample), and N822K (AAT > AAA, in two samples) at 97 months after diagnosis. C. Three types of secondary mutations were detected, including N882K (AAT > AAA, in three abdominal wall tumor samples), D820V (in two mesentery tumor samples), and Y823D (in two mesentery tumor samples) at 109 months after diagnosis (sample 49c).

Additionally, we also observed the temporal heterogeneity of secondary mutations in progressive GISTs. We obtained multiple tumor samples at different time points of progression from eight patients (Cases 49–56 in Supplementary Table S2). No secondary mutations were detected in any of the samples from 3 patients, including 1 patient with primary wild-type GIST (Case 54) at the time of first diagnosis, one with primary *KIT* exon 9 insertion mutation (Case 56), and one with primary mutation in *KIT* exon 11 W557_V559 > C (Case 55). All the 3 types reportedly have a low sensitivity to imatinib.^22-24^ Among the other 5 patients, different secondary mutations were detected in the first and second progressive tumors from different progressive sites in Cases 50 and 51. In Cases 49, 52, and 53, secondary mutations were detected only in the later progressive samples. In a typical patient (Case 49, Supplementary Table S2), no secondary mutation was detected in the first progressive retroperitoneal tumor (sample 49a) at 61 months after diagnosis (Figure 3A), although 4 types of secondary mutations, including D820G, N822Y, N822K (AAT > AAG), and N822K (AAT > AAA), were detected in 6 progressive tumors in the abdominal cavity (sample 49b) at 97 months after diagnosis (Figure 3B). At 109 months after diagnosis, 3 types of secondary mutations were detected, including N882K (AAT > AAA) which was also detected in sample 49b from the second progressive tumors, as well as D820V and Y823D that had never been detected in the first and second progressive tumors (Figure 3C).

### Comparisons of Sanger and NGS in progressive GIST

NGS was performed in 7 patients, with tissue samples obtained from 6 patients and cfDNA samples from one patient (Supplementary Table S2). Among the four cases with single progressive tumors, NGS detected the same primary and secondary mutations as those seen by Sanger sequencing. Besides, some additional gene alterations were detected by NGS in the individual case, such as *PTEN* copy number loss (Case 46, Supplementary Table S2). For the 2 cases (Cases 44 and 49) with multiple progressive tumors, because only 1 sample was tested by NGS, although NGS detected the same secondary mutation as Sanger sequencing for this sample, NGS could not detect the other secondary mutations from the other samples. For Case 43, NGS based on cfDNA only detected D820Y secondary mutation, whereas Sanger sequencing detected D820Y in the tumor from the abdominal wall and other secondary mutations in the retroperitoneal tumor (V654A and S821Y) and recurrent tumor in situ (S821Y and N822K). The results indicated that Sanger sequencing had an advantage in detecting more secondary mutations from multiple tissue samples.

## Discussion

In the present study, we enrolled 69 patients with progressive GISTs who were admitted at our center from June 2003 to March 2023. Compared with previous reports,^5,25,26^ our study showed that progressive GISTs were more frequently observed in younger patients, in patients with the small intestine as the primary tumor site, and in patients with a high risk of developing tumor progression at the initial diagnosis. The mutation rates of *KIT* exon 9 insertion or *KIT* exon 11 deletions were higher in GISTs with progression than in contemporaneous GISTs without progression. Our results suggest strengthening the monitoring of young patients with primary tumors in the small intestine and those with high-risk tumors or *KIT* exon 9 insertion or *KIT* exon 11 deletions at time of diagnosis to ensure early detection of potential tumor progression and timely treatment. Furthermore, these patients may undergo long-term targeted adjuvant therapy to delay tumor progression, as reported by Nishida et al^27^ who examined high-risk GISTs. Additionally, we found that the patients with primary GISTs in the small intestine, those with primary *KIT* exon 11 deletion mutation, and those with continued targeted therapy tended to have progressive tumors in the mesentery and pelvic cavity, indicating that these anatomical sites should be given more attention during follow-up examinations.

Previous studies have reported that approximately half of patients with progressive GIST present with secondary mutations within 2 years after achieving a partial response or at least a stable disease and that the secondary mutation types influence the therapeutic effects of successive treatments.^2,28^ Thus, we need to identify whether patients with progressive GISTs have developed secondary mutations as well as determine the types of these secondary mutations. In our cohort, secondary mutations were detected in 41.1% of patients. Secondary mutations were more frequent in patients with a targeted therapy history and continued targeted therapy until surgery as well as in patients with primary *KIT* exon 11 mutations, which were consistent with the results of previous studies.^11,14,15,17^ Additionally, our results showed that large progressive tumors or progressive tumors located in the chest cavity/pleura, small intestine, abdominal cavity, and mesentery had a higher positivity rate of secondary mutations, indicating that these tumors might be an appropriate sample for detecting secondary mutations.

Importantly, the present study not only demonstrated the intertumoral heterogeneity of secondary mutations but also revealed the intratumoral and temporary heterogeneities of secondary mutations in progressive GISTs. Namløs et al.^29^ detected 10 samples from a case with acquired resistance, with two samples having two types of secondary mutations, similar to our study findings demonstrating two types of secondary mutations in a single tumor. In addition to the finding of multiple secondary mutations in a single tumor, we also found that secondary mutations were detected only in some parts of a single tumor. Moreover, during disease progression, different secondary mutation statuses might also exist at different points in time, similar to the case of our patient whose secondary mutation was detected only in the second and third progressive tumors but not in the first progressive tumor. Thus, extensive sampling might allow clinicians to discover more secondary mutations in progressive GISTs to guide the subsequent tumor therapy.

Sanger sequencing can test multiple samples, thereby more secondary mutations can be detected at a lower cost. Although NGS can detect the same secondary mutations as those detected by Sanger sequencing in the same sample, it is unable to detect other secondary mutations from other tumors that were detected by Sanger sequencing. Contrarily, NGS revealed other gene variations, rather than *KIT*/*PDGFRA* mutations alone, revealing the mechanism of TKI resistance and contributing to the identification of other potential therapeutic targets. Liquid biopsy was considerably useful in detecting secondary mutations, especially for advanced GISTs in which obtaining tumor tissues is challenging. However, the cfDNA samples frequently showed positive results in the patients with metastatic or larger tumor and false negative results in other patients.^29^ Moreover, all cfDNA studies need to rule out false positive results originating from clonal hematopoiesis, which is a natural aging process in which healthy cells of hematopoietic origin can acquire mutations.^30^ In the present study, all 69 patients had a chance of surgery or biopsy after first or second-line treatment, and only one patient performed the liquid biopsy. As expected, although different secondary mutations were present in multiple tumors in different locations, only the secondary mutation from the distant metastatic tumor was detected by liquid biopsy. Therefore, Sanger sequencing was preferable, as it could detect multiple lesions; therefore, testing for secondary mutations could be performed sufficiently, which could ultimately guide the targeted therapies.

Furthermore, patients with progressive tumors without secondary mutations also need to be monitored. Researchers and clinicians have recognized that some patients with high-risk GISTs need a continuous and long-term treatment with imatinib, and an interruption of imatinib treatment leads to disease progression without secondary mutations.^3,27,31^ In our study, among the 22 progressive patients with an interruption of targeted therapy for a period of time before surgery or biopsy, only one patient showed a secondary mutation. Indeed, the relapse rate is <2% per year during adjuvant imatinib treatment for the patients with imatinib-sensitive GIST, but it obviously increases after imatinib therapy is stopped, probably owing to the re-entry of the quiescent cells into a proliferative state.^32-35^ Kang et al reported^31^ that the re-introduction of imatinib achieved an effective tumor control in the patients who developed disease progression after the interruption of imatinib treatment. The abovementioned data suggested that disease progression in the patients with interruption of targeted therapy might not result from secondary mutations and could benefit from the resumption of the previous medicine rather than treatment with later-line drugs. Therefore, an accurate assessment of gene mutations for progressive GISTs is necessary, as this influences the subsequent treatment of the patients.

## Conclusion

Our study underscored the clinicopathologic characteristics of progressive GISTs at the time of initial diagnosis and progression, allowing us to identify the patients with a relatively high risk of disease progression, thereby facilitating timely and appropriate treatment and monitoring. In addition, we demonstrated the intertumoral, intratumoral, and temporal heterogeneities of secondary mutations in progressive GISTs; these data are useful for improving the positivity rate of detecting secondary mutations and for initiating optimal treatments. Nevertheless, due to the limited number of cases analyzed, multicenter studies are needed in the future to confirm our findings.

## Supplementary Material

oyaf110_suppl_Supplementary_Tables_S1-S2

## Data Availability

The data that support the findings of this study are available from the corresponding author upon reasonable request.

## References

[1] Patel SR , Reichardt P. An updated review of the treatment landscape for advanced gastrointestinal stromal tumors. Cancer. 2021;127:2187-2195. https://doi.org/10.1002/cncr.33630 33974733 PMC8252111

[2] Dermawan JK , Rubin BP. Molecular pathogenesis of gastrointestinal stromal tumor: a paradigm for personalized medicine. Annu Rev Pathol. 2022;17:323-344. https://doi.org/10.1146/annurev-pathol-042220-021510 34736340

[3] Klug LR , Khosroyani HM, Kent JD, Heinrich MC. New treatment strategies for advanced-stage gastrointestinal stromal tumours. Nat Rev Clin Oncol. 2022;19:328-341. https://doi.org/10.1038/s41571-022-00606-4 35217782 PMC11488293

[4] Serrano C , Marino-Enriquez A, Tao DL, et al Complementary activity of tyrosine kinase inhibitors against secondary kit mutations in imatinib-resistant gastrointestinal stromal tumours. Br J Cancer. 2019;120:612-620. https://doi.org/10.1038/s41416-019-0389-6 30792533 PMC6462042

[5] Blay JY , Kang YK, Nishida T, von Mehren M. Gastrointestinal stromal tumours. Nat Rev Dis Primers. 2021;7:22. https://doi.org/10.1038/s41572-021-00254-5 33737510

[6] Namlos HM , Zaikova O, Bjerkehagen B, et al Use of liquid biopsies to monitor disease progression in a sarcoma patient: a case report. BMC Cancer. 2017;17:29. https://doi.org/10.1186/s12885-016-2992-8 28061772 PMC5219677

[7] Cao Z , Li J, Sun L, et al GISTs with NTRK gene fusions: a clinicopathological, immunophenotypic, and molecular study. Cancers (Basel). 2022;15:105. https://doi.org/10.3390/cancers15010105 36612101 PMC9817796

[8] Miselli FC , Casieri P, Negri T, et al c-Kit/PDGFRA gene status alterations possibly related to primary imatinib resistance in gastrointestinal stromal tumors. Clin Cancer Res. 2007;13:2369-2377. https://doi.org/10.1158/1078-0432.CCR-06-1745 17438095

[9] Nishida T , Takahashi T, Nishitani A, et al; Japanese Study Group on GIST. Sunitinib-resistant gastrointestinal stromal tumors harbor cis-mutations in the activation loop of the KIT gene. Int J Clin Oncol. 2009;14:143-149. https://doi.org/10.1007/s10147-008-0822-y 19390946

[10] Desai J , Shankar S, Heinrich MC, et al Clonal evolution of resistance to imatinib in patients with metastatic gastrointestinal stromal tumors. Clin Cancer Res. 2007;13:5398-5405. https://doi.org/10.1158/1078-0432.CCR-06-0858 17875769

[11] Lim KH , Huang MJ, Chen LT, et al Molecular analysis of secondary kinase mutations in imatinib-resistant gastrointestinal stromal tumors. Med Oncol. 2008;25:207-213. https://doi.org/10.1007/s12032-007-9014-2 18488160

[12] Liegl B , Kepten I, Le C, et al Heterogeneity of kinase inhibitor resistance mechanisms in GIST. J Pathol. 2008;216:64-74. https://doi.org/10.1002/path.2382 18623623 PMC2693040

[13] Antonescu CR , Besmer P, Guo T, et al Acquired resistance to imatinib in gastrointestinal stromal tumor occurs through secondary gene mutation. Clin Cancer Res. 2005;11:4182-4190. https://doi.org/10.1158/1078-0432.CCR-04-2245 15930355

[14] Nishida T , Kanda T, Nishitani A, et al Secondary mutations in the kinase domain of the KIT gene are predominant in imatinib-resistant gastrointestinal stromal tumor. Cancer Sci. 2008;99:799-804. https://doi.org/10.1111/j.1349-7006.2008.00727.x 18294292 PMC11158696

[15] Debiec-Rychter M , Cools J, Dumez H, et al Mechanisms of resistance to imatinib mesylate in gastrointestinal stromal tumors and activity of the PKC412 inhibitor against imatinib-resistant mutants. Gastroenterology. 2005;128:270-279. https://doi.org/10.1053/j.gastro.2004.11.020 15685537

[16] Wardelmann E , Merkelbach-Bruse S, Pauls K, et al Polyclonal evolution of multiple secondary KIT mutations in gastrointestinal stromal tumors under treatment with imatinib mesylate. Clin Cancer Res. 2006;12:1743-1749. https://doi.org/10.1158/1078-0432.CCR-05-1211 16551858

[17] Heinrich MC , Corless CL, Blanke CD, et al Molecular correlates of imatinib resistance in gastrointestinal stromal tumors. J Clin Oncol. 2006;24:4764-4774. https://doi.org/10.1200/JCO.2006.06.2265 16954519

[18] Du J , Wang S, Wang R, et al Identifying secondary mutations in Chinese patients with imatinib-resistant gastrointestinal stromal tumors (GISTs) by next generation sequencing (NGS). Pathol Oncol Res. 2020;26:91-100. https://doi.org/10.1007/s12253-019-00770-6 31758409

[19] Smith BD , Kaufman MD, Lu WP, et al Ripretinib (DCC-2618) is a switch control kinase inhibitor of a broad spectrum of oncogenic and drug-resistant KIT and PDGFRA variants. Cancer Cell. 2019;35:738-751.e9. https://doi.org/10.1016/j.ccell.2019.04.006 31085175

[20] Chen LL , Holden JA, Choi H, et al Evolution from heterozygous to homozygous KIT mutation in gastrointestinal stromal tumor correlates with the mechanism of mitotic nondisjunction and significant tumor progression. Mod Pathol. 2008;21:826-836. https://doi.org/10.1038/modpathol.2008.46 18488000

[21] Lasota J , vel Dobosz AJ, Wasag B, et al Presence of homozygous KIT exon 11 mutations is strongly associated with malignant clinical behavior in gastrointestinal stromal tumors. Lab Invest. 2007;87:1029-1041. https://doi.org/10.1038/labinvest.3700628 17632543

[22] Cho J , Kang GH, Kim KM, Park J, Shim YM. Aggressive gastrointestinal stromal tumour of the oesophagus with homozygous KIT exon 11 deletion mutation. Pathology (Phila). 2012;44:260-261. https://doi.org/10.1097/PAT.0b013e32834e42f5 22437744

[23] Nannini M , Urbini M, Astolfi A, Biasco G, Pantaleo MA. The progressive fragmentation of the KIT/PDGFRA wild-type (WT) gastrointestinal stromal tumors (GIST). J Transl Med. 2017;15:113. https://doi.org/10.1186/s12967-017-1212-x 28535771 PMC5442859

[24] Guo T , Hajdu M, Agaram NP, et al Mechanisms of sunitinib resistance in gastrointestinal stromal tumors harboring KITAY502-3ins mutation: an in vitro mutagenesis screen for drug resistance. Clin Cancer Res. 2009;15:6862-6870. https://doi.org/10.1158/1078-0432.CCR-09-1315 19861442 PMC2783687

[25] Soreide K , Sandvik OM, Soreide JA, et al Global epidemiology of gastrointestinal stromal tumours (GIST): a systematic review of population-based cohort studies. Cancer Epidemiol. 2016;40:39-46. https://doi.org/10.1016/j.canep.2015.10.031 26618334

[26] Li JX , Sun L, Zhao S, et al Differences in clinicopathological features, gene mutations, and prognosis between primary gastric and intestinal gastrointestinal stromal tumors in 1061 patients. Zhonghua Wei Chang Wai Ke Za Zhi. 2023;26:346-356. https://doi.org/10.3760/cma.j.cn441530-20220531-00234 37072312

[27] Nishida T , Sato S, Ozaka M, et al; STAR ReGISTry Investigators. Long-term adjuvant therapy for high-risk gastrointestinal stromal tumors in the real world. Gastric Cancer. 2022;25:956-965. https://doi.org/10.1007/s10120-022-01310-z 35672526

[28] Heinrich MC , Maki RG, Corless CL, et al Primary and secondary kinase genotypes correlate with the biological and clinical activity of sunitinib in imatinib-resistant gastrointestinal stromal tumor. J Clin Oncol. 2008;26:5352-5359. https://doi.org/10.1200/JCO.2007.15.7461 18955458 PMC2651076

[29] Namlos HM , Boye K, Mishkin SJ, et al Noninvasive detection of ctDNA reveals intratumor heterogeneity and is associated with tumor burden in gastrointestinal stromal tumor. Mol Cancer Ther. 2018;17:2473-2480. https://doi.org/10.1158/1535-7163.MCT-18-0174 30097488

[30] Gomez-Peregrina D , Garcia-Valverde A, Pilco-Janeta D, Serrano C. Liquid biopsy in gastrointestinal stromal tumors: ready for prime time? Curr Treat Options Oncol. 2021;22:32. https://doi.org/10.1007/s11864-021-00832-5 33641024

[31] Kang YK , Kim HD, Kim HJ, et al Interruption of imatinib in advanced gastrointestinal stromal tumor after prolonged imatinib maintenance in the absence of gross tumor lesions. Gastric Cancer. 2023;26:604-613. https://doi.org/10.1007/s10120-023-01377-2 36884149

[32] Joensuu H , Eriksson M, Sundby Hall K, et al Survival outcomes associated with 3 years vs 1 year of adjuvant imatinib for patients with high-risk gastrointestinal stromal tumors: an analysis of a randomized clinical trial after 10-Year follow-up. JAMA Oncol. 2020;6:1241-1246. https://doi.org/10.1001/jamaoncol.2020.2091 32469385 PMC7260691

[33] Casali PG , Le Cesne A, Velasco AP, et al Final analysis of the randomized trial on imatinib as an adjuvant in localized gastrointestinal stromal tumors (GIST) from the EORTC soft tissue and bone sarcoma group (STBSG), the australasian gastro-intestinal trials group (AGITG), UNICANCER, french sarcoma group (FSG), italian sarcoma group (ISG), and spanish group for research on sarcomas (GEIS)(*). Ann Oncol. 2021;32:533-541. https://doi.org/10.1016/j.annonc.2021.01.004 33482247

[34] Raut CP , Espat NJ, Maki RG, et al Efficacy and tolerability of 5-year adjuvant imatinib treatment for patients with resected intermediate- or high-risk primary gastrointestinal stromal tumor: the PERSIST-5 clinical trial. JAMA Oncol. 2018;4:e184060. https://doi.org/10.1001/jamaoncol.2018.4060 30383140 PMC6440723

[35] Joensuu H , Wardelmann E, Sihto H, et al Effect of KIT and PDGFRA mutations on survival in patients with gastrointestinal stromal tumors treated with adjuvant imatinib: an exploratory analysis of a randomized Clinical Trial. JAMA Oncol. 2017;3:602-609. https://doi.org/10.1001/jamaoncol.2016.5751 28334365 PMC5470395

