# Supplementary material for: Clinicopathological characteristics of progressive gastrointestinal stromal tumors and heterogeneity analyses of secondary mutations
Source: Oncologist. 2025 May 16;30(5):oyaf110. doi: 10.1093/oncolo/oyaf110 (PMC12082821; doi:10.1093/oncolo/oyaf110)
Supplement: oyaf110_suppl_Supplementary_Tables_S1-S2 [file oyaf110_suppl_supplementary_tables_s1-s2.pdf]

Supplementary Table S1. The sequences of primers used in Sanger sequencing

Gene-Exon	Primer sequences
KIT-exon 9	Forward: 5’-ACTAAACTCATCTGGGCCACC-3’
	Reverse: 5’-TAGACAGAGCCTAAACATCCCC-3’
KIT-exon 11	Forward: 5’-TCCAGAGTGCTCTAATGACTGAG-3’
	Reverse: 5’-GCCCCTGTTTCATACTGACCA-3’
KIT-exon 13	Forward: 5’-ACATCAGTTTGCCAGTTGTGC-3’
	Reverse: 5’-CAGCTTGGACACGGCTTTA-3’
KIT-exon 14	Forward: 5’-CCTTGGGTATTTTTATGGGAGGC-3’
	Reverse: 5’-ACCAGGAAGACTCCTTTGAATGC-3’
KIT-exon 17	Forward: 5’-TGGTTTTCTTTTCTCCTCCAACC-3’
	Reverse: 5’-TGCAGGACTGTCAAGCAGAG-3’
KIT-exon 18	Forward: 5’-CTGTTCAATTTTGTTGAGCTTCTGA-3’
	Reverse: 5’-TGTCTTTGGCAAGGATCATTTTACC-3’
PDGFRA-exon 12	Forward: 5’-GTGCACTGGGACTTTGGTAAT-3’
	Reverse: 5’-AAAAGGGAGTCTTGGGAGGT-3’
PDGFRA-exon 14	Forward: 5’-GGTAGCTCAGCTGGACTGAT-3’
	Reverse: 5’-GGATGGAGAGTGGAGGATTT-3’
PDGFRA-exon 18	Forward: 5’-AGACAGCTCCAAGTGCCACCA-3’
	Reverse: 5’-ACCGAATCTCTAGAAGCAACA-3’

Supplementary Table S2. Detailed information of the 67 samples from 56 cases of progressive GIST in which secondary mutations were tested

No.	Sex /Age*	Primary tumors: Primary Site	Primary tumors: Risk degree	Primary tumors: Primary mutation	Months after diagnosis	Targeted therapy#	Progressive tumors: Progressive site§	Progressive tumors: No. (tested samples/available results)	Progressive tumors: Secondary mutation‖
1	F/41	jejunum	High	V560_Y578del	103	Yes/NA	abdominal cavity (1)	1/1	N822I (1)
2	F/40	stomach	High	W557_K558del	69	Yes/Yes	retroperitoneal (1)	1/1	N822K (1)
3	M/56	stomach	Intermediate	W557_V559>C	49	Yes/Yes	progression in situ (1)	2/2	V654A (2)
4	F/62	stomach	High	V559_N566>D	50	Yes/Yes	abdominal cavity (1)	3/3	V654A (3)
5	M/60	stomach	High	K558_V560del	29	Yes/Yes	progression in situ (1)	2/2	V654A (2)
6	M/43	stomach	Low	E554_K558del	70	Yes/Yes	abdominal wall (1)	1/0	ND
							mesentery (23)	2/2	V654A (1)
							peritoneum (20)	2/2	V654A (2)
7	F/44	small intestine	NA	L576P	31	Yes/Yes	abdominal cavity (5)	5/3	V654A (1)
							abdominal wall (1)	1/0	ND
							colorectal (1)	3/0	ND
							pelvic cavity (2)	2/1	V654A (1)
8	M/67	small intestine	High	K642E	74	Yes/Yes	progression in situ (8)	3/0	ND
							retroperitoneal (2)	2/2	ND
							abdominal wall (1)	1/1	ND
							peritoneum (27)	14/14	N822K (1)
9	M/46	small intestine	Intermediate	Y503_F504insAY	87	Yes/No	liver (4)	4/1	ND
							pelvic cavity (2)	3/3	ND
							adjacent structures (3)	5/2	N822K (1)
10	M/60	stomach	NA	E554_K558del	16	Yes/Yes	progression in situ (2)	2/2	T670I (2)
							retroperitoneal (1)	2/2	ND

							pelvic cavity (1)	2/1	ND
11	M/48	rectum	Intermediate	W557_V559>F	118	Yes/Yes	liver (3)	3/3	V654A (1)
12	M/55	ileum	Low	V560_Y578del	60	Yes/Yes	pelvic cavity (2)	4/4	D820H (1)
							mesentery (1)	5/5	C809G (5)
							omentum majus (7)	7/7	ND
13	F/57	stomach	High	W557_K558del	108	Yes/Yes	abdominal cavity (1)	5/1	Y823D (1)
							abdominal wall (12)	8/7	Y823D (5)
							mesentery (10)	10/6	D820Y (1), Y823D (2)
14	M/45	jejunum	High	Y503_F504insAY	172	Yes/Yes	mesentery (4)	5/4	D820G (2), D820V (2)
15	M/60	stomach	NA	W557_K558del	35	Yes/Yes	progression in situ (1)	2/1	Y823D and A829P (1)[†]
							liver (2)	2/2	A829P (2)
16	M/62	rectum	High	W557_V559>C	97	Yes/Yes	small intestinal (7)	6/5	D820Y (2), D820V (2), Y823D (1)
17	M/60	small intestine	Intermediate	Y503_F504insAY	11	No/No	progression in situ (1)	1/1	ND
							mesentery (1)	1/1	ND
							peritoneum (1)	1/1	ND
							jejunum (1)	3/1	ND
18	F/41	stomach	Intermediate	W557R	61	Yes/Yes	liver (2)	2/2	ND
19	F/50	small intestine	High	Wild Type	95	No/No	progression in situ (1)	1/1	ND
							pelvic cavity (1)	2/0	ND
							mesentery (2)	1/1	ND
20	M/60	stomach	High	Wild Type	64	Yes/No	progression in situ (1)	3/2	ND
21	M/47	small intestine	Intermediate	Y503_F504insAY	22	No/No	liver (2)	3/2	ND
22	M/56	stomach	High	K550_V555>QL	83	Yes/No	retroperitoneal (4)	5/4	ND
							abdominal wall (5)	5/5	ND
23	F/28	small intestine	Intermediate	Y503_F504insAY	18	No/No	progression in situ (4)	4/4	ND
							peritoneum (3)	3/3	ND

24	F/64	stomach	Intermediate	V560G	71	Yes/NA	progression in situ (2)	2/1	ND
							retroperitoneal (6)	6/4	ND
25	F/30	rectum	Low	E554_K558del	86	Yes/No	progression in situ (1)	1/1	ND
26	F/56	duodenum	High	V556_P573del	32	No/No	liver (3)	3/3	ND
27	M/46	jejunum	Intermediate	Wild Type	110	No/No	progression in situ (1)	2/1	ND
							abdominal cavity (1)	1/1	ND
							abdominal wall (1)	1/0	ND
							omentum (2)	2/0	ND
28	F/58	duodenum	High	Y503_F504insAY	26	No/No	liver (2)	3/2	ND
29	F/56	small intestine	High	M552_E554>I	83	Yes/Yes	abdominal wall (1)	2/1	ND
							pelvic cavity (1)	4/2	ND
							mesentery (1)	1/1	ND
							omentum (3)	3/2	ND
30	F/53	stomach	Very low	D579del	139	No/No	progression in situ (2)	3/3	ND
31	F/52	retroperitoneal	High	W557_V559>C	151	Yes/No	progression in situ (1)	2/1	ND
32	M/57	stomach	High	W557_K558del	78	Yes/No	abdominal cavity (1)	1/1	ND
							omentum (1)	1/1	ND
33	F/57	mesentery	High	Y503_F504insAY	24	Yes/No	colorectal (15)	2/2	ND
34	F/62	small intestine	High	Y503_F504insAY	55	Yes/Yes	adjacent structures (1)	1/1	ND
							mesentery (1)	2/1	ND
35	M/67	abdominal cavity	High	D842V	12	No/No	progression in situ (5)	8/8	ND
36	M/60	duodenum	High	V569_L576del	74	No/No	progression in situ (2)	2/1	ND
							adjacent structures (3)	5/3	ND
							mesentery (1)	1/1	ND
							omentum (6)	6/3	ND

37	F/47	small intestine	Intermediate	Wild Type	17	Yes/Yes	pelvic cavity (20)	4/3	ND
38	M/64	stomach	High	W557_K558del	33	Yes/No	mesentery (1)	2/2	ND
39	M/77	stomach	High	W557_K558>FP	22	No/No	progression in situ (1)	1/1	ND
40	M/49	stomach	NA	*PDGFRA* D842V	23	Yes/Yes	pelvic cavity (1)	1/1	ND
41	M/60	stomach	Intermediate	*PDGFRA* D842V	24	Yes/Yes	abdominal cavity (1)	1/1	ND
							liver (1)	1/1	ND
42	F/17	stomach	NA	Wild Type	56	Yes/Yes	liver (2)	1/1	ND
43	M/69	small intestine	High	W557_K558del	48	Yes/Yes	abdominal wall (2)	1/1	D820Y (1)
							progression in situ (1)	2/2	S821Y (1), N822K (1)
							retroperitoneal (6)	3/3	V654A (2), S821Y (1)
43-NGS							cfDNA	1/1	D820Y
44	M/67	jejunum	High	W557_K558del	29	Yes/Yes	progression in situ (1)	1/1	S821_N822insR (1)
							liver (1)	1/1	Y823S (1)
							mesentery (5)	6/6	S821_N822insR (2)
							adjacent structures (1)	1/1	S821_N822insR (1)
44-NGS							liver	1/1	Y823S<br>*RB1*: c.939+2T>C; *MAX*: R35H
45	M/66	rectum	High	K558_V559>N	84	Yes/Yes	pelvic cavity (1)	1/1	D820Y (1)
45-NGS							pelvic cavity (1)	1/1	D820Y
46	M/62	small intestine	High	V560D	93	Yes/Yes	retroperitoneal (1)	1/1	ND
46-NGS							retroperitoneal (1)	1/1	*PTEN* copy number loss
47	M/43	stomach	NA	I571_D572insE	29	Yes/Yes	liver (1)	1/1	D820Y (1)

47-NGS							liver (1)	1/1	D820Y<br>*TP53*: R175H
48	M/28	small intestine	NA	Q556_V559>H	203	Yes/Yes	stomach (1)	1/1	V654A (1)
48-NGS							stomach (1)	1/1	V654A
49a	M/53	abdominal cavity	High	K558_V559>N	61	Yes/NA	retroperitoneal (1)	3/2	ND
49b					97	Yes/Yes	progression in situ (6)	6/6	D820G (1), N822Y (2), N822K (3) ‡
49c					109	Yes/Yes	mesentery (2)	3/3	D820V (2), Y823D (1)
							abdominal wall (19)	3/3	N822K (3) ‡
49c-NGS							mesentery (1)	1/1	Y823D<br>*LRP1B*: Y4201C; *ERBB4*: D871N
50a	F/38	stomach	Intermediate	L576_D579del	152	Yes/Yes	retroperitoneal (1)	1/1	K826N (1)
50b					182	Yes/Yes	liver (3)	1/1	N822K (1)
51a	M/55	small intestine	High	W550_W557>IL	150	Yes/Yes	abdominal wall (2)	3/3	D820Y (2), N822K (1)
51b					158	Yes/Yes	liver (1)	2/2	D820Y (2)
52a	F/58	stomach	High	V559D	32	No/No	liver (1)	2/2	ND
52b					66	Yes/Yes	lung (1)	1/1	V654A (1)
52c					86	Yes/Yes	chest wall (2)	2/2	V654A (2)
53a	M/45	stomach	Low	W557_K558del	79	Yes/No	thoracic cavity (1)	1/1	ND
53b					99	Yes/Yes	thoracic cavity (1)	1/1	T670I (1)
54a	M/69	stomach	High	Wild Type	13	Yes/Yes	mesentery (1)	1/1	ND
54b					25	Yes/Yes	omentum (21)	3/3	ND
							abdominal wall (1)	1/1	ND
							mesentery (1)	1/1	ND
							progression in situ (1)	1/1	ND

54c					32	Yes/Yes	progression in situ (1)	3/3	ND
							omentum (3)	2/2	ND
55a	F/50	stomach	High	W557_V559>C	42	Yes/No	abdominal cavity (1)	2/2	ND
55b					88	Yes/No	abdominal cavity (1)	1/1	ND
56a	M/65	mesentery	High	Y503_F504insAY	18	Yes/Yes	liver (3)	1/1	ND
56b					22	Yes/Yes	liver (1)	2/1	ND
							adjacent structures (1)	1/1	ND
							omentum (15)	2/1	ND
							small intestinal (6)	3/2	ND
							progression in situ (1)	1/1	ND
							abdominal wall (1)	1/1	ND

Abbreviations: F: female; M: male; NA: biopsy samples or referral patients in which the risk degree of the tumor cannot be assessed; ND: not detected;
*: age at diagnosis; #: history of targeted therapy/continued targeted therapy until surgery or biopsy; §: involved anatomical site and number of tumors during disease progression; |: secondary mutation type and number of samples for each secondary mutation; †: Y823D and A829P were simultaneously detected in the same sample in Case 16; ‡: although the N822K mutation was detected in several specimens from both samples 48b and 48c, the detailed bases were different, including c.2466T>A in two specimens from sample 48b and all three specimens from sample 48c and c.2466T>G in one specimen from sample 48b.
All listed samples were exanimated for KIT mutations in exons 9, 11, 13, 14, 17, and 18 and PDGFRA mutations in exons 12, 14, and 18. Exons not specifically listed are wild-type.
